# Addressing substance abuse and violence in substance use disorder treatment and batterer intervention programs

**DOI:** 10.1186/1747-597X-7-37

**Published:** 2012-09-07

**Authors:** Christine Timko, Helen Valenstein, Patricia Y Lin, Rudolf H Moos, Gregory L Stuart, Ruth C Cronkite

**Affiliations:** 1 Center for Health Care Evaluation, Department of Veterans Affairs Health Care System and Stanford, University Medical Center, Palo Alto, CA, USA; 2 Department of Psychology, University of Washington, Seattle, WA, USA; 3 Department of Psychology, University of Tennessee-Knoxville, Knoxville, TN, USA; 4 Center for Health Care Evaluation, VA Health Care System (152-MPD), 795 Willow Road, Menlo Park, CA, 94025, USA

**Keywords:** Substance use disorder, Substance abuse treatment policy, Batterer intervention, Intimate partner violence, Treatment integration, Service centralization

## Abstract

**Background:**

Substance use disorders and perpetration of intimate partner violence (IPV) are interrelated, major public health problems.

**Methods:**

We surveyed directors of a sample of substance use disorder treatment programs (SUDPs; N=241) and batterer intervention programs (BIPs; N=235) in California (70% response rate) to examine the extent to which SUDPs address IPV, and BIPs address substance abuse.

**Results:**

Generally, SUDPs were not addressing co-occurring IPV perpetration in a formal and comprehensive way. Few had a policy requiring assessment of potential clients, or monitoring of admitted clients, for violence perpetration; almost one-quarter did not admit potential clients who had perpetrated IPV, and only 20% had a component or track to address violence. About one-third suspended or terminated clients engaging in violence. The most common barriers to SUDPs providing IPV services were that violence prevention was not part of the program’s mission, staff lacked training in violence, and the lack of reimbursement mechanisms for such services. In contrast, BIPs tended to address substance abuse in a more formal and comprehensive way; e.g., one-half had a policy requiring potential clients to be assessed, two-thirds required monitoring of substance abuse among admitted clients, and almost one-half had a component or track to address substance abuse. SUDPs had clients with fewer resources (marriage, employment, income, housing), and more severe problems (both alcohol and drug use disorders, dual substance use and other mental health disorders, HIV + status). We found little evidence that services are centralized for individuals with both substance abuse and violence problems, even though most SUDP and BIP directors agreed that help for both problems should be obtained simultaneously in separate programs.

**Conclusions:**

SUDPs may have difficulty addressing violence because they have a clientele with relatively few resources and more complex psychological and medical needs. However, policy change can modify barriers to treatment integration and service linkage, such as reimbursement restrictions and lack of staff training.

## 

Substance use disorders and intimate partner violence (IPV) are interrelated, major public health problems. Of men entering substance use disorder treatment programs (SUDPs), approximately 60% have perpetrated IPV [1-4], and of clients in Batterer Intervention Programs (BIPs; programs that treat IPV perpetration), similar proportions have SUDs. For example, of men court-referred to BIPs in Rhode Island, 68% were hazardous drinkers, 53% had an alcohol use disorder, and 31% had a drug use disorder [5]. Accordingly, to improve the quality of care and reduce the harm caused by SUDs and IPV, substance abuse policy researchers are calling for improved linkages between SUD and IPV perpetration treatment systems and programs, as well as better recognition of the cross problem within programs.

This study focused on a sample of programs in California to examine the extent to which SUDPs address IPV in their client population, and BIPs address SUDs among their clients. The study’s design was guided by a modified version of Moos’ (1997) conceptual framework [6] for informing SUD policy through evaluations of treatment programs (Figure 1). This model is broadly consistent with the model of organizational change in addictions treatment outlined by Simpson (2004) [7]. It highlights the role of organizational factors and aggregate client characteristics in shaping the use of specific linkage practices within and across programs and how these factors influence treatment outcomes. Specifically, the model suggests that linkages are shaped by characteristics of the organization, such as size and funding, and the types of clients served [8,9], and that stronger in-program and cross-program linkages are associated with more positive client outcomes [10,11]. However, we lack critical data from SUDPs and BIPs on these domains, particularly the extent to which SUDPs address IPV in their client population and the extent to which BIPs address substance abuse among their clients.

**Figure 1:**
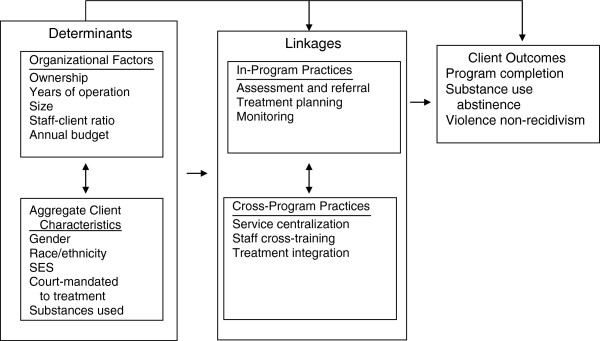
Determinants and outcomes of SUDP and BIP linkages.

## Organizational and client characteristics

Organizational factors that influence service linkages and outcomes include the program’s ownership, years of operation, size, staffing, annual budget, and revenue sources. Organizational factors are important to assess in part because they are associated with treatment services and client outcomes [11,12]. Unfortunately, surveys of SUDPs and BIPs do not yield comprehensive, comparable information on organizational factors. Surveys of SUDPs often combined data on a range of program settings (outpatient, intensive outpatient, short- or long-term residential, inpatient), whereas BIPs do not treat a diagnosed disorder and fall into the category of outpatient only. In addition, available surveys (especially those of BIPs) often had very low response rates and, because some were conducted a number of years ago, may not reflect current program characteristics.

The National Survey of Substance Abuse Treatment Services (N-SSATS), an annual survey of facilities providing substance abuse treatment, reported that most facilities are private, non-profit (58%), and that 60% receive government funding [13]. Private-for-profit, private-nonprofit, and government-funded facilities in the N-SSATS were comparable on the likelihood of offering domestic violence (victim and/or perpetrator) services (32%-38% did so) [14]. The National Drug Abuse Treatment Clinical Trials Network (CTN), in a survey of outpatient, methadone, long-term residential, and inpatient program staff members, found that 36% had a master’s or doctoral degree [15]. The National Treatment Center Study (NTCS) of SUDPs found that 45% of counselors held a master’s degree [16].

A national survey of BIPs found that 43% were private-nonprofit, 48% were private-for-profit, and 9% were publicly owned [17]. Program income was mainly from client fees (74%), with 18% from government sources. In a subsequent national survey of BIPs, 54% of programs were funded exclusively by client payments; 87% relied in part on client fees, and 46% received some funding from another source, such as the government, private donations, or foundations [18]. BIPs varied widely in size, with an average of 195 [17] and 131 [18] clients per year. Dalton reported that most BIP staffs were small, with 85% having at most four full-time workers [17]. However, in contrast to the cited SUDP surveys, which had very high response rates, the BIP survey response rates were low (49% in Dalton [17], 15% in Price & Rosenbaum [18]).

Important client characteristics are indicative of personal needs and resources and include gender, race and ethnicity, socioeconomic status, court mandates to treatment, and types of substances used. Client characteristics are also likely to be associated with treatment services and outcomes. Again, however, there is a lack of data to directly compare client characteristics between SUDPs and BIPs.

The N-SSATS found that most clients in facilities treating substance abuse were male (68%) and White (63%), with 21% Black and 14% Hispanic. In addition, 43% of clients had abused both alcohol and other drugs, 39% abused drugs only, and 18% abused alcohol only. Furthermore, 43% of clients had co-occurring mental health disorders [13]. In the NTCS, 39% of clients were women, 28% were referred to treatment from the legal system, and 43% were on probation or parole [16]. In the CTN, 10% of outpatient clients were homeless, and 34% were on probation [15]. Finally, in the National Drug Abuse Treatment System Survey of outpatient programs, 53% of clients were described as potentially benefiting from HIV testing and counseling [8]. These findings suggest that many SUDP clients have a complex array of problems that could make a program emphasis on violence perpetration more important but also more difficult. The BIP surveys found that about 90% of BIP clients were men, and 89% were court mandated to attend the program [17,18]. BIP clients were mainly White (58%), with 21% Black and 17% Hispanic [17]. Although income was not assessed, BIP clients were described as being of low socioeconomic status [18].

## In- and cross-program practices

Given the high co-occurrence of SUDs and IPV perpetration, in-program practices of careful assessment, appropriate referral and intervention planning, and monitoring of at-risk clients are important for addressing both problems [5,19-22]. However, in a survey of SUD and IPV (victim and batterer) programs in Illinois (35% and 47% response rates, respectively), formal screening for the cross problem was rare and unsystematic [23]. A national survey of SUDPs (61% response rate) and IPV programs found that only 60% of SUDPs screened for, and only 49% treated, IPV perpetration, and only 46% had a formal referral arrangement with an IPV (victim and/or batterer) program [12]. In the IPV programs (75% response rate, but only 31% of responding programs provided batterer intervention services), 58% screened for, and 19% treated, SUDs, and 47% had a formal referral arrangement with a SUDP. These findings provide some evidence of a lack of implementation of recommended in-program practices [24,25]; however, the surveys were conducted more than a decade ago and tended to have relatively low response rates.

Although there is relatively little definitive information, some evidence suggests that referrals to BIPs are infrequent (17%) even among SUDP clients with a pretreatment–year history of IPV perpetration, because providers inconsistently assessed IPV perpetration, or used assessment strategies that were not evidence-based [26]. For example, assessments for IPV perpetration may consist of a single question (e.g., have you hit your spouse) that allows clients to say no honestly even though they had engaged in IPV (e.g., kicked, hit with an object, used a weapon). According to Schumacher and colleagues [26], SUD treatment providers often do not provide referrals even when IPV perpetration is discovered, and only 13% of clients receiving a BIP referral followed through and enrolled, due in part to a lack of monitoring of referrals.

Staff members in SUDPs often lack expertise in violence screening and treatment, and BIP staff members often do not have training in SUD assessment and treatment [23]. In Collins and Spencer’s survey, 54% of SUDPs, and 26% of IPV programs, had a staff member attending to the cross-problem [12]. Even then, staff expertise was rarely based on formal training; for example, a BIP staff member in recovery was considered an expert in SUD treatment.

Treatments for both SUDs and IPV perpetration within the same program may be viewed as incompatible. SUDs are seen as the primary problem in SUDPs, and IPV perpetration is seen as primary in BIPs. SUDPs may view IPV perpetration as a problem not requiring intervention in its own right. In this view, the battering will stop if the substance use stops [12], or at least SUDs must be addressed before other areas of dysfunction can be addressed [27].

## Client outcomes

Program completion rates are estimated at 42% in outpatient SUDPs nationally [13], and 50%-75% in BIPs [28-31]. Program completion is important because it is associated with better client outcomes in both systems of care. Specifically, clients who complete substance abuse treatment are more likely to achieve abstinence [32,33], and those who complete batterer intervention may be more likely to refrain from additional violence perpetration [34,35].

## Study aims

Our aims were to study a sample of SUDPs and BIPs in California to be able to fully describe and directly compare programs. We examined programs’ organizational (for example, do SUDPs and BIPs differ on numbers and training levels of staff members available to clients) and aggregate client characteristics (do SUDPs have clients with fewer resources and more health needs). We also compared programs on policies about admitting clients with the cross problem, assessing and monitoring the cross problem, and referring treated clients who engage in the cross problem. For example, do SUDPs admit potential clients who have perpetrated IPV, and do BIPs admit potential clients who have misused alcohol and other drugs?

We examined within-program treatment of the cross problem: what kinds of services, if any, do SUDPs offer for IPV perpetration, and do BIPs provide substance abuse services? Among programs that do not offer cross-problem services, what are the barriers to doing so? Do program directors believe that integrating substance abuse and violence treatment may give clients a higher likelihood of achieving positive outcomes? Improving our knowledge of how SUDPs and BIPs manage IPV perpetration and SUDs, respectively, will help policymakers gauge the extent to which inadequate service linkage is occurring and make recommendations accordingly. It will also help program managers and providers establish effective linkages and implement interventions better to reduce substance abuse and violence, thereby improving the quality of care in these settings.

## Methods

### Sample

The sampling frame was all BIPs in the state, using a current listing by the California State Auditor. Specifically, the listing consisted of BIPs approved by criminal justice agencies (via an annual on-site review examining adherence to applicable statutes and regulations) within each county jurisdiction. Although BIPs are not typically described as providing treatment for a diagnosed disorder, all would fall in the category of outpatient care. Within each of the state’s 58 counties, the same number of SUD outpatient treatment programs as BIPs was randomly selected, using SAMHSA’s Substance Abuse Treatment Facility Locator. Considering sampling with respect to county reduced the likelihood of extraneous factors (e.g. judicial practices; availability of health, social, and other services) affecting results. We confirmed the accuracy of contact information and requested surveys from directors of 339 SUD treatment programs and 339 Batterer Intervention Programs. By using a sequence of follow-up procedures to target non-responders [36], we obtained completed surveys from 241 SUDPs and 235 BIPs (response rates of 71% and 69%, respectively). All procedures were approved by Stanford University’s Institutional Review Board.

### Survey

Where possible, survey items were drawn from previously used measures, i.e. the Residential Substance Abuse and Psychiatric Programs Inventory [37,38]; and Bennett and Lawson’s Illinois [23], and Collins and Spencer’s [12], surveys of SUDPs and IPV-related programs. An initial draft of the survey was pretested with 6 program directors (3 SUDP, 3 BIP) selected at random from the potential participant pool. Feedback from the pretest was used to finalize the survey.

The survey ascertained program organizational characteristics, aggregate client characteristics, within-program practices (assessment and referral, treatment planning, and monitoring of IPV in SUDPs and of SUDs in BIPs) and cross-program practices (service centralization, staff cross-training, and treatment integration). It also assessed program-level outcomes among clients leaving the program in the past year: rates of program completion (“What percentage of the clients who left your program in the past 12 months completed the program [i.e. did not drop out or were terminated prematurely]?”), substance use abstinence (calculated from, “What percentage of the clients who left your program in the past 12 months were using alcohol and/or drugs at discharge?”), and IPV perpetration abstinence (from, “What percentage of the clients who left your program in the past 12 months were perpetrating IPV or battering at discharge?”). Other specific items are described in the Results section.

Evidence supports the validity of outcomes at the program (or client aggregate) level in that they are relatively stable (i.e. are not sensitive to changes in the individual making the report or to turnover in the client or staff population) and have convergent and discriminate validity [39,40]. More generally, research shows that SUDP directors, including those serving offenders, provide valid and reliable data on program practices and their determinants and outcomes [37,38,41,42].

### Data analysis

We compared SUDPs to BIPs mainly using t-tests for continuous variables, and chi-square tests for categorical variables.

## Results

### SUDPs

On average, the length of treatment obtained by SUDP clients was about six months (mean=191.5 days, SD=165.3). The majority (75% or more) of SUDPs offered the SUD-related services of individual counseling, group counseling, continuing care, or case management. Roughly one-third to one-half of programs offered marital or family counseling, mental health treatment, 12-step meetings on-site, job counseling, or help with housing. Fewer than one in four programs offered peer counseling, rides or subsidies for public transportation to and from the program, child care, smoking cessation, legal services, detoxification, or methadone and/or buprenorphine or other medications for SUDs. On a scale from 1=none, to 10=very strong, on average, programs were rated as placing more emphasis on abstinence from alcohol and drugs (mean=9.5, SD=1.2) than on harm reduction (mean=5.7, SD=3.7).

Also on a scale of 1 (none) to 10 (very strong), on average, programs primarily emphasized a cognitive-behavioral (M=8.7, SD=2.0), twelve-step (M=8.0, SD=2.7), or motivational interviewing (M=7.7, SD=2.4) treatment model. Less emphasized were Matrix, social, community reinforcement, psychodynamic, family, and dual diagnosis treatment models (means range from 5.8 to 6.8), and even lesser used were psychosocial rehabilitation, contingency management, and medical treatment models (means from 2.7 to 4.8).

### BIPs

On average, the length of treatment obtained by BIP clients was one year (mean=365.4 days, SD=22.2). The majority of BIPs offered counseling for IPV perpetration in groups for men only (98.3%) and for women only (74.4%), but very few offered groups for men and women together (2.1%). The majority offered group or individual anger management counseling (60.7%), but fewer offered individual IPV perpetration counseling (39.7%). Parenting classes were offered by 40.3% of BIPs, but 12.0% or less offered marital or family counseling, peer counseling, legal services, child care, mutual-help groups for perpetrators on-site, or rides or subsidies for public transportation to and from the program. On the 1 (none) to 10 (very strong) scale, on average, programs primarily emphasized a cognitive-behavioral (M=8.5, SD=2.1) or psychoeducational (M=8.1, SD=2.4) treatment model, and put less emphasis on the Duluth, family systems, psychodynamic, or psychodrama model (means from 3.5 to 6.7).

### Organizational characteristics

On average, compared to BIPs, SUDPs had been operating longer, served more clients, and had more paid staff, including those providing direct service to clients (Table 1). SUDPs had more staff members per 100 clients. SUDPs also had a larger annual budget, with the largest percentage of the budget coming from government sources. BIPs were supported mainly by client fees. SUDPs were mainly not-for-profit, whereas BIPs were more evenly split between not-for-profit and for-profit ownership status. More than 75% of SUDPs and BIPs were located in urban or suburban areas.

**Table 1:** Organizational characteristics of SUDPs (N = 241) and BIPs (N = 235)

	**SUDPs**	**BIPs**	**t**
	**(Mean, SD)**	**(Mean, SD)**	
Number of:			
Years program has been operating	20.3 (11.6)	15.2 (8.1)	5.46***
Unique clients served, past year	378.6 (1263.6)	164.9 (347.8)	2.43*
Paid staff	9.8 (9.9)	4.3 (3.6)	8.08***
Paid staff providing direct service	7.0 (7.8)	3.7 (3.0)	6.18***
Paid, direct care staff with professional degrees	3.1 (6.7)	2.3 (2.3)	1.53
Number of staff per 100 clients	11.7 (40.9)	5.4 (6.8)	2.25*
Operating budget (annual, in 1000 s)	$786 ($1,262)	$132 ($254)	5.91***
Percent of budget from:			
Client fees	34.2 (41.0)	88.7 (27.5)	15.71***
Government	57.6 (42.8)	7.3 (22.6)	14.67***
Private sources	5.5 (16.9)	2.3 (11.3)	2.24*
Other sources	2.6 (13.0)	1.6 (10.6)	.85
	SUDPs	BIPs	X^2^
	(percent, N)	(percent, N)	
Type of ownership			28.35***
Public	16.3(39)	6.0(14)	
Private, not for profit	58.3(141)	48.1(113)	
Private, for profit	25.4(61)	46.0(108)	
Type of area			.56
Urban	50.8(123)	53.6(126)	
Suburban	27.9(67)	27.7(65)	
Rural	21.3(51)	18.7(44)	

*p < .05 **p < .01 ***p < .001.

### Aggregate client characteristics

Both SUDPs and BIPs served a preponderance of men, but SUDPs had a lower proportion of men than BIPs did, as well as a lower proportion of Hispanic/Latino clients, and a higher proportion of White clients (Table 2). On average, compared to BIPs, SUDPs served more youth and middle-aged clients. They also served a somewhat more disadvantaged clientele; that is, fewer clients were married/partnered or employed and they tended to have less income and were more likely to be homeless. In addition, SUDP clients had more severe problems in that they were more likely to have both alcohol and drug use disorders, dual substance use and other mental health disorders, and HIV + status.

**Table 2:** Aggregate client characteristics of SUDPs (N = 241) and BIPs (N = 235)

	**SUDPs**	**BIPs**	**t**
	**(Mean %, SD)**	**(Mean %, SD)**	
Male	63.8 (21.6)	84.1 (16.1)	-11.26***
Race and ethnicity:			
Hispanic/Latino	29.8 (24.9)	40.1 (27.2)	-4.18***
Black, non-Hispanic	12.5 (24.9)	15.5 (17.4)	1.90
White, non-Hispanic	48.2 (29.2)	37.2 (26.6)	4.17***
Other	9.7 (20.2)	7.4 (16.2)	1.32
Age			
Under age 21	10.3 (13.6)	7.4 (7.9)	2.42*
21-40 years old	55.3 (21.5)	62.6 (18.5)	-3.46***
41-55 years old	27.2 (17.5)	23.3 (14.2)	2.30*
Over age 55	6.7 (7.5	6.6 (6.8)	.22
Married/partnered	47.2 (23.1)	65.8 (22.0)	-8.51***
College graduate	17.1 (20.1)	13.3 (16.0)	1.88
Employed	42.6 (27.4)	58.3 (23.1)	-5.69***
Annual income			
$20,000 or less	57.5 (35.0)	40.8 (29.8)	4.78***
$20,001-$40,000	22.6 (20.0)	38.1 (22.9)	-6.77***
$40,001-$80,000	15.6 (22.2)	16.6 (15.7)	-.48
$80,001 or more	4.6 (10.8)	4.2 (8.7)	.32
Homeless	12.0 (17.0)	7.4 (12.0)	2.86**
Have alcohol use disorder only	15.7 (15.2)	23.6 (17.1)	-5.04***
Have drug use disorder only	23.6 (22.0)	12.5 (17.1)	6.81***
Have both alcohol and drug use disorders	60.6 (29.7)	32.9 (26.3)	10.39***
Dually diagnosed	51.4 (27.3)	16.3 (18.7)	2.65***
HIV+	4.7 (6.6)	2.5 (5.7)	13.36***
Mandated to program by	53.1 (35.3)	90.7 (15.9)	-14.55***
Criminal Justice System			
Arrested due to substance abuse	72.6 (29.7)	47.4 (29.5)	8.06***
Men arrested for IPV perpetration	14.7 (21.0)	85.2 (21.8)	-24.84***
Women arrested for IPV perpetration	7.6 (17.4)	71.3 (36.1)	-17.86***

*p < .05 **p < .01 ***p < .001.

Slightly over one-half of SUDP clients were mandated to treatment by the criminal justice system, but 91% of BIP clients were similarly mandated. SUDP clients were more likely to have been arrested for reasons related to substance abuse than were BIP clients, but even so, almost one-half of BIP clients had been arrested for substance abuse-related reasons. As expected, compared to BIPs, SUDPs had lower proportions of men and women who had been arrested for IPV perpetration.

### In-program practices

#### Assessment

Less than one-quarter of SUDPs had a policy regarding the assessment of IPV perpetration among people seeking treatment, whereas over one-half of BIPs had a policy regarding assessment of substance abuse among potential clients (Table 3). For both SUDPs and BIPs, when programs had a policy, it was mainly to require assessment of the cross problem.

**Table 3:** In-program practices regarding assessment and referral of SUDPs (N=241) and BIPs (N=235)

	**SUDPs**	**BIPs**	**X**^**2**^
**Assessment**	**(% yes, N)**	**(% yes, N)**	
Program has a policy:			
On assessing the cross problem	22.0 (53)	54.5 (128)	54.59***
Of programs having a policy (N=181; 53 SUDPs, 128 BIPs):			
Policy requires assessment of cross problem	73.6	91.4	9.84***
Cross problem is assessed at least sometimes among people seeking help from program	68.0 (168)	93.6 (22)	58.84***
Of programs assessing the cross problem at least sometimes (N=384; 164 SUDPs, 220 BIPs):			
Every client is assessed	66.7	86.3	20.97***
A standard, published scale is used	15.2	43.6	37.12***
Current pattern is assessed	69.0	94.2	34.37***
Current severity is assessed	62.8	89.0	28.79***
Problem history is assessed	92.9	91.1	.32
Past severity is assessed	59.3	82.2	18.77***
**Referral: Alcohol Abuse and IPV Perpetration**	SUDPs	BIPs	X^2^
If potential client has cross problem, program does not admit the client	24.1 (58)	27.2 (64)	.63
**Referral: Drug Abuse and IPV Perpetration**			
If potential client has the cross problem, program does not admit the client	24.1 (58)	25.1 (59)	.07
Follows up on referrals to determine if potential clients obtained help	51.6 (124)	61.1 (144)	3.04*

*p < .05 **p < .01 ***p < .001.

SUDPs were less likely than BIPs to require assessment of the cross problem, and to assess it at least sometimes among potential clients (Table 3). Among programs that assessed the cross problem at least sometimes, SUDPs were less likely than BIPs to assess every potential client, and to use a standard, published scale for assessments. When assessing the cross problem among potential clients, SUDPs were less likely than BIPs to assess the current pattern and severity, and the past severity, of the problem.

#### Referral

A total of 24.1% of SUDPs did not admit potential clients who had perpetrated IPV (Table 3). The survey pretest had suggested that BIPs may address alcohol misuse differently from drug misuse among potential clients. However, similar proportions did not admit potential clients with alcohol and drug abuse (27.2% and 25.1% of BIPs, respectively). Over one-half of both types of programs followed up with potential clients to determine whether they obtained help from programs to which they were referred, but SUDPs were less likely to do so than BIPs.

Among SUDPs that did not admit clients because of a known violence history, 78% provided the client with a referral; of these, referrals were most often to a BIP (78%) but may have been to the criminal justice system, such as a probation officer (29%), or to another SUDP (16%) (not tabled). Among SUDPs that did admit clients despite a known violence history, 93% made an additional requirement, recommendation, or referral; these clients were most often referred to the program’s own anger management component (50%), and less often to a BIP (42%), the program’s own batterer intervention component (21%), or the criminal justice system (9%).

Among BIPs that did not admit clients because of a known alcohol abuse history, 92% provided the client with a referral. Of these programs, 70% sometimes referred the client to a SUDP, 41% to the criminal justice system, and 5% to another BIP. BIPs’ referral patterns of clients with drug abuse were quite similar to those for alcohol abuse.

Among BIPs that admitted clients despite a known alcohol abuse history, 94% made an additional requirement, recommendation, or referral. Of these, 60% may refer the client to a SUDP to attend simultaneously, 24% may require or recommend participation in the same program’s substance abuse treatment component, 26% may mandate attendance at 12-step mutual-help groups, and 26% may refer the client to the criminal justice system. Again, results pertaining to drug abuse were quite similar to those for alcohol abuse.

#### Treatment planning

SUDPs and BIPs were comparable on the likelihood of offering at least one service targeting the cross problem; almost two-thirds of programs did so (Table 4). SUDPs were less likely than BIPs to offer group counseling, but more likely to offer rides and legal services, with regard to the cross problem.

**Table 4:** In–program practices regarding treatment planning of SUDPs (N=241) and BIPs (N=235)

	**SUDPs**	**BIPs**	**X**^**2**^
	**(% yes, N)**	**(% yes, N)**	
Any service offered for cross problem	64.3 (155)	63.0 (148)	.09
Offers cross problem service of:			
Individual counseling	23.3 (56)	29.2 (69)	2.96
Group counseling	28.6 (69)	56.2 (132)	37.49***
Marital/family counseling	18.1 (44)	15.0 (35)	.79
Mutual-help groups	4.2 (10)	6.4 (15)	1.15
Peer counseling	3.8 (9)	6.9 (16)	2.26
Rides to and from other program	6.7 (16)	1.3 (3)	9.85**
Legal services	5.9 (14)	2.1 (5)	4.42*
Program has a specific component or track to treat the cross problem	20.5 (49)	47.7 (112)	39.74***
Program requires clients with cross problem to make a contract to refrain from the behavior	55.8 (134)	75.1 (176)	16.45***
Program does not provide services for cross problem (N=173; 86 SUDPs, 87 BIPs) because:			
Services for other problem are not part of this program’s main mission or focus	71.0	51.1	13.27***
Staff lack expertise in other problem	41.5	22.0	13.81***
No mechanisms exist to reimburse services for other problem	26.1	20.6	1.35
Services for other problem are better provided independently from services for this problem	22.7	41.1	12.41***
Services for other problem are not required for licensing, certification, or accreditation	19.3	44.0	22.63***
Clients will not have cross problem after they are helped for this problem	.6	0	1.18

*p < .05 **p < .01 ***p < .001.

SUDPs were less likely to have a component or track to address IPV perpetration than were BIPs to have a component or track to address substance abuse (Table 4). Of SUDPs with a component or track addressing IPV perpetration, 49% required the component for all clients as a regular part of treatment, 24% required it only for clients with a history of IPV perpetration, and 27% made it optional for clients (not tabled). Also, of SUDPs with a component or track addressing IPV perpetration, 97% provided it in the same location where the SUDP was located.

Of BIPs with a component or track addressing substance abuse, 37% required the substance abuse component for all clients as a regular part of treatment, 19% required it only for clients with a history of substance abuse, 28% required it only for clients who have a history of substance abuse and were court-referred to treatment, and 16% made it optional for clients (not tabled). Also, of BIPs with a component or track addressing substance abuse, 85% provided substance abuse treatment in the same location where the BIP was located. As shown in Table 4, when clients were identified as having the cross problem, SUDPs were less likely to require a no-violence contract than were BIPs to require a no-substance use contract.

Of programs that did not provide services for the cross problem, SUDPs were more likely than BIPs to state that cross services were not part of the program’s mission and that staff lack needed expertise (Table 4). SUDPs were less likely than BIPs to state that service provision is better when the two problems of SUD and violence are treated independently, and that services for the other problem are not required for licensure or certification.

#### Monitoring

Only 31% of SUDPs had a policy requiring the monitoring of IPV perpetration among clients, whereas 64% of BIPs had a policy requiring monitoring of substance abuse among clients (Table 5). SUDPs also were less likely than BIPs to monitor the cross problem at least sometimes among clients. In addition, among programs that monitored the cross problem, SUDPs were less likely than BIPs to monitor every client, to use a standard scale, and to make notes in the client’s record regarding monitoring.

**Table 5:** In-program practices regarding monitoring of SUDPs (N=241) and BIPs (N=235)

	**SUDPs**	**BIPs**	**X**^**2**^
**Program has a policy:**	**(% yes, N)**	**(% yes, N)**	
On monitoring the cross problem	30.6 (74)	64.3 (151)	42.93***
Of programs having a policy (N=225; 74 SUDPs, 151 BIPs), policy:			
Requires monitoring of the cross problem	100	100	N/A
Cross problem is monitored at least sometimes	47.9 (115)	83.3 (196)	68.04***
Of programs monitoring the cross problem at least sometimes (N=311; 115 SUDPs, 196 BIPs):			
Every client is monitored	28.1	53.3	19.12***
A standard, published scale is used	9.7	23.9	10.12***
Notes are made in program record	71.1	82.1	4.96*
**Monitoring: Alcohol Abuse and IPV Perpetration**			
When a client is known to engage in cross problem during treatment, program:			
Suspends or terminates client	35.7 (86)	39.6 (93)	.76
**Monitoring: Drug Abuse and IPV Perpetration**			
When a client is known to engage in cross problem during treatment, program:			
Suspends or terminates client	35.7 (86)	40.0 (94)	.94
Follows up on referrals to determine if terminated clients obtained help	42.9 (103)	42.4 (100)	.95
Program collects follow-up information from former clients	53.8 (130)	17.6 (41)	69.50***

*p < .05 **p < .01 ***p < .001.

Among SUDPs monitoring only selected clients for IPV perpetration during treatment (that is, they monitored violence at least sometimes, but not for every client), 42% monitored only clients with a violence history, and 58% had another practice or no program-wide practice (staff members made their own decisions) (not tabled). In most of these programs (60%), monitoring occurred with the frequency that staff members determined to be appropriate for each client.

Among BIPs monitoring selected clients for substance abuse during treatment, 37% monitored only clients with a history of substance abuse, and 63% had another practice or no program-wide practice. In most of these programs (51%), staff members determined the frequency of monitoring clients.

#### Consequences of cross problem identification during treatment

Just over one-third of SUDPs suspended or terminated clients who were known to perpetrate violence during treatment, and about the same proportion of BIPs suspended or terminated clients who were known to use substances during program participation (Table 5). Among SUDPs that suspended or terminated clients because of a known violence event, 87% provided the client with a referral (not tabled). The most common referrals were to the criminal justice system (61%); other referrals were to a BIP (28%), to an anger management program (24%), or to another SUDP (11%).

Almost all (97%) SUDPs that continued to treat clients despite a known violence event made an additional recommendation regarding violence-related interventions. Programs tended to recommend simultaneous attendance at a BIP (40%), an anger management program (38%), or a batterer intervention component in their program (15%). A total of 22% may refer the client to the criminal justice system, and 8% may give the client a warning or put the client on probation.

Among BIPs that suspended or terminated clients because of known alcohol use, 94% provided the client with a referral. Of these, 77% referred the client to the criminal justice system (e.g., probation officer); programs sometimes referred the client to a SUDP (32%), and one program sometimes referred clients to another BIP. Results pertaining to program responses to drug use were quite similar to those for alcohol use.

Among BIPs that continued to treat clients despite known alcohol use during treatment, virtually all (99%) made an additional recommendation regarding substance abuse interventions. Of these programs, 47% may refer the client to a SUDP to attend simultaneously, 38% may refer the client to the criminal justice system, 26% may require or recommend participation in the same program’s substance abuse treatment component, and 14% may give the client a warning or put the client on probation. These results pertaining to alcohol use closely resembled those for drug use.

Less than one-half of SUDPs and BIPs followed up on referrals to determine if clients whose treatment was terminated due to the cross problem obtained help to which they were referred (Table 5). Over one-half of SUDPs collected follow-up information from former clients, whereas only 18% of BIPs did so.

### Cross-program practices

#### Service centralization

SUDPs were more likely than BIPs to have a single phone number for potential clients seeking help for both substance abuse and violence problems, but were less likely to have help available for both problems at a single location (Table 6).

**Table 6:** Cross-program practices of SUDPs (N=241) and BIPs (N=235)

	**SUDPs**	**BIPs**	**X**^**2**^
**Service Centralization**	**(% yes, N)**	**(% yes, N)**	
To obtain help for both problems, potential clients can:			
Call only one phone number	56.8 (137)	45.5 (107)	5.39*
Complete only one set of intake procedures	26.0 (63)	24.6 (58)	.10
Obtain both at a single location	32.9 (79)	46.6 (110)	8.88**
Have their program records shared with another of the cross type	20.6 (50)	23.0 (54)	.40
Program has arrangements with another program to refer clients to:			
Of the same type	2.9 (7)	3.4 (8)	.09
Of the cross type	17.8 (43)	21.3 (50)	.89
Of programs that have arrangements with the cross type (N=93; 43 SUDPs, 50 BIPs):			
Formal arrangement	3.8	12.1	2.78
Informal arrangement	61.5	47.0	2.49
**Staff Training**			
Direct care staff are informed about addressing cross problem:			29.10***
All	54.0 (130)	79.3 (186)	
Some	24.5 (59)	14.5 (34)	
None	21.5 (52)	6.2 (15)	
Direct care staff’s training in cross problem:			86.90***
All are required to have formal training	12.9 (31)	45.4 (107)	
Some (certain positions) required to have formal training	24.1 (58)	23.8 (56)	
Some have informal training	32.3 (78)	24.7 (58)	
No staff members have formal or informal training	30.6 (74)	6.2 (15)	
**Treatment Integration**			
Program philosophy of best way to serve clients with both problems:			
Help for both problems should be obtained at the same time, but in separate treatment programs	60.2 (145)	56.5 (133)	.64
Help for both problems should be integrated into one treatment program	31.0 (75)	29.7 (70)	.09
Substance abuse treatment should be completed first, followed by batterer intervention or anger management	9.7 (23)	19.0 (45)	7.87**
Batterer intervention or anger management should be completed first, followed by substance abuse treatment	.5 (1)	.4 (1)	.00
Potential clients seeking help for both problems:			21.72***
Are permitted to obtain cross help while in this program	61.0 (147)	72.8 (171)	
No usual practice; depends on client and staff member	33.5 (81)	22.4 (53)	
Should complete this program before obtaining cross help	4.6 (11)	.4 (1)	
Must obtain cross help before this program	.9 (2)	4.4 (10)	
Would more linkages, cooperation between SUDP and BIP communities benefit your clients?			9.26*
Definitely no	1.7 (4)	0 (0)	
Probably no	5.2 (12)	5.6 (13)	
Probably yes	47.2 (114)	39.2 (92)	
Definitely yes	45.9	45.9 (111)	55.2 (130)
Barriers to your program being linked and cooperating with the cross type of program:			
Differences in treatment philosophy between communities	9.9 (24)	12.9 (30)	1.02
Lack of substance abuse treatment training in BIPs	24.2 (58)	16.3 (38)	4.44*
Lack of batterer intervention training in SUDPs	36.3 (87)	23.6 (55)	8.84**
Lack of cross type of program in this area	19.3 (47)	9.0 (21)	10.12***
SUDPs not reimbursed for batterer intervention	31.8 (76)	15.0 (35)	18.32***
BIPs not reimbursed for substance abuse	10.8 (26)	29.2 (69)	24.89***
SUDPs and BIPs compete for clients	4.0 (10)	9.0 (21)	4.73*

*p < .05 **p < .01 ***p < .001.

Relatively few SUDPs and BIPs had an arrangement to refer clients to another program of the same type or of the cross type (Table 6). Of programs that had a referral arrangement with the cross type of program, arrangements were more likely to be informal than formal in both types of programs.

#### Staff cross-training

SUDPs were less likely than BIPs to require that all direct care staff be informed about assessing, treating, and monitoring the cross problem (Table 6). They were also less likely to require all direct care staff to have formal training in the cross problem, and more likely to have no formal or informal training requirements.

#### Treatment integration

Each program director was asked the extent to which the program integrates treatment of the cross problem into its core function (0=not at all, 1=slightly, 2=moderately, 3=strongly, 4=very strongly). On average (not tabled), SUDPs were less integrated than were BIPs (Means [SDs]=1.2 [1.2] and 2.3 [1.1], respectively; t=10.17, p < .001).

When asked about the best way to serve clients with both problems, both SUDP and BIP directors most commonly answered that help for both problems should be obtained simultaneously, but in separate programs (Table 6). Slightly less than one-third of both sets of program directors stated that help for both problems should be integrated into one program. SUDP directors were less likely than BIP directors to state that substance abuse treatment should be completed first, followed by batterer intervention or anger management, but virtually no program director responded that batterer intervention should be completed before substance abuse treatment. In addition, no program directors stated that treatment for the cross problem was unnecessary or even harmful (not tabled).

Regarding clients seeking help for both substance abuse and violence perpetration, most SUDPs and BIPs permitted (did not prohibit) clients to obtain help for the cross problem while receiving services within the program (Table 6). However, more BIPs permitted simultaneous help for substance abuse than SUDPs permitted simultaneous help for violence perpetration. Roughly one-third of SUDPs and one-quarter of BIPs did not have a standardized practice regarding clients’ help for the cross problem; rather, staff members made their own decisions depending on the client. Very few SUDPs and BIPs required that clients with both problems complete the program first, or recommended obtaining help for the cross problem first. A lower proportion of SUDPs than BIPs stated a definite yes that more linkages between the SUDP and BIP communities would be helpful to clients (Table 6). Of note, more than 90% of SUDP and BIP directors stated that more linkages between programs would probably or definitely be beneficial to their clients.

Regarding barriers to linkages between the SUDP and BIP communities, SUDP directors were more likely than BIP directors to endorse the barriers of lack of substance abuse training in BIPs, and lack of batterer intervention training in SUDPs, as well as the lack of a cross type of program in the same geographical area (Table 6). SUDP directors were more likely to endorse the barrier of SUDPs not being reimbursed for batterer intervention, whereas BIP directors were more likely to endorse the barrier of BIPs not being reimbursed for substance abuse treatment. Relatively few programs endorsed competition for clients as a linkage barrier, but SUDPs were less likely to do so.

### Outcomes

Among clients leaving programs in the past year, SUDPs, in comparison to BIPs, had lower rates of program completion (Means [SDs]=59.8% [23.1%] vs. 67.8% [25.8%]; Mann–Whitney U=18882.5, z=−4.401, p < .001), but higher rates of clients who were abstinent from from perpetrating IPV (97.4 % [5.2%] vs. 91.3% [15.2%]; Mann–Whitney U=6671.0, z=−3.546, p < .001).

## Discussion

### SUDPs and IPV perpetration

Generally, substance use disorder treatment programs were not addressing co-occurring violence in a formal and comprehensive way when we considered the in-program practices of client intake, treatment, and monitoring. Few SUDPs (only 39 of 241 surveyed; 16%) had a policy requiring potential clients to be assessed for violence perpetration, although most (68%) assessed potential clients’ violence perpetration at least sometimes. Almost one-quarter of SUDPs did not admit potential clients who perpetrated IPV, and only about one-half followed up on referrals to determine that help was obtained. Anecdotal evidence suggested that non-admission of IPV perpetrators was due to staff perceptions that these clients needed mental health services beyond the scope of the SUDP. Only 20% of SUDPs had a specific component or track to address violence, and only about one-quarter offered individual or group counseling for IPV perpetration.

The most common reasons SUDPs did not provide IPV services were that violence prevention was not part of the program’s mission, and staff members lacked training in violence prevention and management. Services outside SUDPs’ focus are often prohibited by reimbursement policies [43]. That is, SUDPs are often not allowed to bill for IPV perpetration services and so do not provide them [12]. However, such policies, as well as lack of staff training, are both remediable barriers [44]. For example, regarding staff training, SUDPs could adopt an IPV prevention toolkit comprised of a DVD to illustrate clinical tools, a laminated counselor guide, and worksheets and wallet cards for clients to retain key points [45].

In SUDPs, monitoring violence among admitted clients appeared to be emphasized more than assessing violence among potential clients. Specifically, almost twice as many programs (N=75; 31%) had a policy requiring monitoring clients for violence. Still, only 48% of programs monitored violence at least sometimes. About one-third of SUDPs suspended or terminated clients known to engage in violence, and only 43% followed up on referrals to determine if terminated clients obtained help.

### BIPs and substance abuse

In contrast, BIPs appeared to be addressing substance abuse in a relatively formal and comprehensive way. One-half (117 of 235 BIPs surveyed) had a policy requiring potential clients to be assessed for substance abuse, and 94% assessed potential clients’ substance abuse at least sometimes. Similar to SUDPs, one-quarter of BIPs did not admit potential clients with the cross problem, but a higher proportion (61%) followed up on referrals to determine if potential clients obtained help. In addition, 56% of BIPs offered group, and 29% offered individual, counseling related to substance abuse, and almost one-half of BIPs had a specific component or track to address substance abuse. SUD services were not provided in BIPs most often because they were not part of the program’s mission, and were not required for licensing, certification, or accreditation. Criteria for approving programs could be extended to include substance abuse assessment, treatment, and/or ongoing monitoring [43,46].

Supporting the idea that BIPs were more fully addressing substance abuse than SUDPs were addressing violence, we also found that 64% of BIPs (N=151) had a policy requiring monitoring of substance abuse among clients, and 83% monitored substance abuse at least sometimes. However, 40% suspended or terminated clients known to abuse substances, and similar to SUDPs, only 42% followed up on referrals to determine if terminated clients obtained help. BIPs may focus more on substance abuse because of widespread recognition by BIP directors that substance abuse among their clients is a predictor of program dropout [47,48].

It is possible that SUDPs placed less emphasis on addressing violence because their clients had fewer resources (marriage, job, income, housing) and more severe and numerous problems needing attention (both alcohol and drug use disorders, dual substance use and other mental health disorders, HIV + status). The difficulties of treating clients with multiple, complex problems may have competed with and dissuaded consideration of violence perpetration. Supplementary analyses (not yet presented) supported this hypothesis in that programs with higher proportions of clients who were unmarried, unemployed, and low-income, with both alcohol and drug use disorders, and both substance use and mental health disorders, were less likely to assess for and monitor the cross problem. On the other hand, SUDPs had a higher staff-patient ratio to address these challenges of the client population. Higher staff-patient ratios are indicative of higher quality care [49], but the larger number of staff members in SUDPs may make requiring all staff to be trained in violence prevention and management less practical; smaller staff sizes in BIPs may make cross training in substance abuse more feasible, despite possible higher costs of training per staff member should a trainer be brought in to the program. Notably, almost one-third of SUDPs did not have even one staff member with formal or informal training in IPV. More broadly, the NTCS identified large training gaps among addiction counselors that raise concerns about the integrity with which care is delivered [50], particularly to clients with co-occurring disorders and problems [51]. Shortages of trained staff may be more severe in rural areas [52] where some SUDPs and BIPs were located. The provision of toolkits mentioned previously, in combination with web-based resources, may ease the difficulties of staff training in the cross-problem when programs have large staff numbers or rural locations.

Returning to the finding that SUDPs had clients with fewer resources and more severe problems, SUDPs may complete more frequent and thorough assessments of these problems than BIPs do, because SUDPs are administered by health agencies, and BIPs are administered within the criminal justice system. That is, SUDPs have the primary goal of service provision, whereas BIPs, as justice-related organizations, have the primary goal of public safety [53]. Policy researchers have urged both the addiction treatment and criminal justice systems to move toward a more even balance between public health and public safety through systems integration [53]. To achieve more integration between the SUDP and BIP systems, one step is to define and develop reliable and valid tools to measure such integration, and to institute policies to support integration. In addition, program directors could become experts on cross-problem evidence-based practices that they disseminate effectively to staff, and establish working relationships with cross agencies that encourage staff to collaborate and coordinate efforts [10,53,54]. Other suggestions are to use change teams within programs (e.g., a SUDP would select a change leader, who would gather ideas for addressing violence, focus on clients with a history of IPV perpetration to understand and consider their needs, and evaluate improvements to address violence), and to partner government agencies with multiple programs to support greater access to and implementation of cross services [55]. For example, county probation departments that oversee BIPs could partner with SUDPs within their county to achieve better integration of services for substance abusing individuals who perpetrate IPV.

### Service centralization and treatment integration

We found relatively little evidence that services are centralized for individuals with both substance abuse and violence-related problems. Roughly one-half of SUDPs and BIPs combined were able to help dual-problem clients via a call to only one phone number; SUDPs were more likely than BIPs to offer this aspect of centralization. However, about three-quarters of SUDPs and BIPs required clients with both problems to complete multiple sets of intake procedures. In addition, about three-quarters of SUDPs, and one-half of BIPs, did not provide help for both problems at a single location, despite findings that health care consumers prefer “one stop shopping” for co-occurring needs [56]. Furthermore, about 80% of SUDPs did not share client records with any BIP, and about the same proportion of BIPs did not share client records with any SUDP. Although it is quite common for individuals to need help with both substance abuse and violence perpetration, these results show that services for these two interrelated problems are not centralized to facilitate client access and utilization.

As reviewed by Bennett [52], in practice, substance abuse and IPV perpetration have often been viewed as independent - separate problems with different interventions. Our data suggest that this view is held within the majority of both SUDPs and BIPs in that most directors endorsed the statement that help for both substance abuse and violence should be obtained at the same time, but in separate treatment programs. This is known as parallel treatment. There is some evidence that parallel treatment has positive effects on reducing substance abuse and domestic violence [52]. The disadvantage of parallel substance abuse and batterer programs is that the time and financial commitments may become a burden, engender resistance in clients and their family members, and increase perceived hardship in an already-difficult situation [52]. In addition, without explicit integration of treatment, clients may have difficulty managing the cognitive and affective components of battering intervention treatment during early abstinence. Specifically, individuals in early abstinence often experience memory loss, emotional dysregulation, and poor impulse control [57,58].

Research also suggests that integrated substance abuse and family relationship treatment reduces both substance abuse and family violence for some couples. Our results show that a sizeable minority within both SUDPs and BIPs – about 30% -- endorse having help for both substance abuse and IPV perpetration integrated into one treatment program. In this regard, Behavioral Couples Therapy enhances both abstinence and relationship functioning to promote recovery and reduce violent behavior [59,60]. Similarly, Brannen and Rubin found that for court-ordered men with alcohol problems, participation in a couples group produced greater reductions in physical abuse than did participation in a men’s group [61]. Importantly, that study included components to protect victims’ safety [62]. Couples counseling may be detrimental for victims experiencing the form of IPV known as intimate terrorism (the violence is embedded in a general pattern of the perpetrator attempting to exert control over the partner) rather than situational couple violence, in which specific arguments escalate to violence [63]. In this regard, to ensure victim safety, couples counseling should be considered only after providers have had time to gain confidence that situational couple violence, rather than intimate terrorism, is occurring [64]. Studies support conclusions that for some couples improvements in relationship functioning and substance use outcomes jointly account for reductions in IPV perpetration associated with substance abuse treatment [3].

### Program outcomes

In keeping with having clients with fewer resources and more psychological and medical difficulties, SUDPs had higher attrition rates than BIPs did [65]. Average drop-out rates were 40% in SUDPs, and 32% in BIPs. In both types of programs, about 25%-30% were using substances at program discharge, and under 10% were engaging in partner violence. Increasing client retention rates may serve to improve outcomes [32-35]. One suggestion is to discuss possible barriers to retention with clients, so that they can be addressed. Common barriers are unmet social services needs (help with employment, housing, court hearings, child care) and lack of flexibility in scheduling around work and family requirements [66]. In addition to improving retention rates, we need programs to collect more systematic follow-up information on SUDP, and especially BIP, clients.

### Limitations

This study had strengths and weaknesses. In terms of strengths, we achieved high response rates for this type of survey in samples of SUDPs and BIPs. We collected a substantial amount of useful information from both types of programs. Weaknesses are that we studied programs in only one state using a cross-sectional design with program-level outcomes, to the exclusion of client-level data. We did not independently audit each program; thus, the accuracy of data presented is based on reports by program directors, who may have slightly different responses in some cases than other staff members, such as clinicians [67]. Even so, this dataset provides a useful benchmark against which to assess future efforts to link services addressing substance abuse and violence. In this regard, our survey showed that the great majority of SUDP and BIP directors agreed that more linkages and cooperation between the two communities would benefit clients.

## Conclusions

We found that SUDPs were addressing violence perpetration in a less formal and comprehensive way than BIPs were addressing substance abuse among their clients. More research is needed to determine whether a more comprehensive approach to addressing co-occurring substance abuse and violence contributes to better completion rates and client outcomes. To the extent that such associations exist, the information gathered in this study underscores that more could be achieved in terms of addressing cross problems and establishing linkages between these two types of programs.

More could be done by programs in terms of assessing and referring clients who have the cross problem. At present, only 16% of SUDPs and 50% of BIPs require assessment of the other problem. Individuals who have experienced the cross problem are at risk of not being admitted to the program or for limited follow-up if they are given a referral. Alternative approaches to addressing dual substance abuse and violence problems that ensure clients’ access to services with more systematic follow-up have the potential to improve outcomes for these complex clients.

We identified barriers to better linkages, such as lack of reimbursement and staff training for the cross problem, that are modifiable through changes in policy. In this regard, reimbursement policies could be altered to allow programs to bill for services related to addressing the cross problem, and certification policies could be changed to encourage staff training in the cross problem. To be feasible for implementation, staff policies may require only a subset of staff members, rather than all staff, to be trained in assessment, treatment, and referrals for the cross problem and their follow-through. Together, such policy changes could broaden the pool of staff trained in both types of problems, thus allowing for the enhancement and expansion of the program’s main mission or focus, and, in turn, the provision of appropriate services for clients with dual problems, such as in one integrated program or in separate but linked programs offering parallel services.

Although we noted some important differences between clients in SUDPs and BIPs, there were also some broad similarities of clients in the two types of programs (for example, most were mandated to treatment), suggesting that they may respond well to an integrated treatment approach. In this regard, the commonly used cognitive-behavioral model in both SUDPs and BIPs should ease efforts to integrate treatment. In addition, motivational interviewing, a treatment model emphasized heavily in SUDPs, is increasingly being applied in batterer programs [68], and so may provide a potential path toward more integrated substance abuse-batterer care.

The consequences of failure to address co-occurring substance abuse and violence can be quite severe, including permanent injury or death to victims. Therefore, it behooves us to continue to effect policy change to facilitate treatment integration and service centralization to reduce substance abuse and perpetration of intimate partner violence.

## Competing interests

We, the authors, declare that we have no competing interests.

## Authors’ contributions

CT conceived of and designed the study, oversaw data collection, set up, and analysis; and drafted and revised the manuscript. HV and PL made substantial contributions to the study design; collected, entered, set up, and analyzed the data; and helped to revise the manuscript. RM and GS made substantial contributions to the study design, data collection procedures, and interpretation of results, and helped to revise the manuscript. RC co-conceived, designed, and oversaw the study, and helped to revise the manuscript. All authors read and approved the final manuscript.

## References

[B1] Brown TG Werk A Caplan T Shields N Seraganian P The incidence and characteristics of violent men in substance abuse treatment Addict Behav 1998 2 573 586 9768295 10.1016/s0306-4603(98)00004-5

[B2] Chermack ST Fuller BE Blow FC Predictors of expressed partner and non-partner violence among patients in substance abuse treatment Drug Alcohol Depend 2000 58 43 54 10.1016/S0376-8716(99)00067-8 10669054

[B3] Murphy CM Ting L The effects of treatment for substance use problems on intimate partner violence: a review of empirical data Aggress Violent Beh 2010 15 325 333 10.1016/j.avb.2010.01.006

[B4] O’Farrell TJ Murphy CM Marital violence before and after alcoholism treatment J Consult Clin Psychol 1995 63 256 262 7751486 10.1037//0022-006x.63.2.256

[B5] Stuart GL Moore TM Kahler CW Ramsey SE Substance abuse and relationship violence among men court-referred to batterers’ intervention programs Subst Abuse 2003 24 107 122 10.1080/08897070309511539 12766378

[B6] Moos RH Evaluating treatment environments 1997 New Brunswick: Transaction

[B7] Simpson DD A conceptual framework for drug treatment process and outcomes J Subst Abuse Treat 2004 22 171 182 15450644 10.1016/j.jsat.2004.06.001

[B8] D’Aunno T The role of organization and management in substance abuse treatment: review and roadmap J Subst Abuse Treat 2006 31 221 233 10.1016/j.jsat.2006.06.016 16996385

[B9] Oser CB Knudsen HK Staton-Tindall M Taxman FE Leukefield C The adoption of wraparound services among women-specific and non-women-specific substance abuse treatment programs serving criminal offenders Drug Alcohol Depend 2009 103S S82 S90 19181457 10.1016/j.drugalcdep.2008.12.008 PMC2784607

[B10] Fletcher BW Lehman WEK Wexler HK Melnick G Taxman FS Young DW Measuring collaboration and integration activities in criminal justice and substance abuse treatment agencies Drug Alcohol Depend 2009 103S S54 S64 20088023 10.1016/j.drugalcdep.2009.01.001

[B11] Flynn PM Knight DK Godley MD Knudsen HK Introduction to the special issue on organizational dynamics within substance abuse treatment: a complex human activity system J Subst Abuse Treat 2012 42 109 115 10.1016/j.jsat.2011.10.029 22154828

[B12] Collins JJ Spencer DL Linkage of domestic violence and substance abuse services 1999 Research Triangle Park: Research Triangle Institute

[B13] SAMHSA (Substance Abuse and Mental Health Services Administration) National survey of substance abuse treatment services (N-SSATS)): 2010 2011 Rockville: Substance Abuse and Mental Health Services Administration

[B14] N-SSATS spotlight: Domestic violence services [Internet] SAMHSA National Survey of Substance Abuse Treatment Services (N-SSATS) 2010 Rockville: Substance Abuse and Mental Health Services Administration http://www.oas.samhsa.gov/spotlight/Spotlight020DomesticViolence.pdf.

[B15] McCarty D Fully BE Arfken C Miller M Nunes EV Edmundson E Copersino M Floyd A Forman R Laws R Mcgruder KM Oyama M Prather K Sinclar J Wendt WW Direct care workers in the national drug abuse treatment clinical trials network: characteristics, opinions, and beliefs Psych Serv 2007 58 181 190 10.1176/appi.ps.58.2.181 PMC2861362 17287373

[B16] Roman PM Ducharme LJ Knudsen HK Patterns of organization and management in private and public substance abuse treatment programs J Subst Abuse Treat 2006 31 235 243 10.1016/j.jsat.2006.06.017 16996386

[B17] Dalton B What’s going on out there? A survey of batterer intervention programs J Aggression Maltreat Trauma 2007 15 59 74

[B18] Price BJ Rosenbaum A Batterer intervention programs: a report from the field Violence Vict 2009 24 757 770 10.1891/0886-6708.24.6.757 20055213

[B19] Bennett L O’Brien P Effects of coordinated services for drug-abusing women who are victims of intimate partner violence VAW 2007 13 395 411 10.1177/1077801207299189 17420517

[B20] Fazzone PA Holton JK Reed BG TIP 25: Substance abuse treatment and domestic violence 1997 Rockville: SAMHSA, CSAT

[B21] Leonard K Domestic violence and alcohol J Subst Use 2001 6 235 247 10.1080/146598901753325075

[B22] National Quality Forum National voluntary consensus standards for the treatment of substance use conditions: evidence-based treatment practices 2007 Washington DC: National Quality Forum

[B23] Bennett L Lawson M Barriers to cooperation between domestic violence and substance abuse programs Fam Soc 1994 75 277 286

[B24] Klostermann KC Substance abuse and intimate partner violence Subst Abuse Treat Prev Policy 2006 1 1 24 10.1186/1747-597X-1-1 PMC1564385 16925813

[B25] Klostermann KC Fals-Stewart W Intimate partner violence and alcohol use Aggress Violent Behav 2006 11 587 597 10.1016/j.avb.2005.08.008

[B26] Schumacher JA Fals-Stewart W Leonard KE Domestic violence treatment referrals for men seeking alcohol treatment J Subst Abuse Treat 2003 24 279 283 10.1016/S0740-5472(03)00034-5 12810149

[B27] Goldkamp J The Role of drug and alcohol abuse in domestic violence and its treatment: dade county’s domestic violence court experiment 1996 Washington, DC: U.S. Department of Justice, National Institute of Justice

[B28] Dalton B Batterer characteristics and treatment completion J Interpers Violence 2001 16 1223 1238 10.1177/088626001016012001

[B29] Daly JE Pelowski S Predictors of dropout among men who batter Violence Vict 2000 15 137 160 11108498

[B30] Rooney J Hanson RK Predicting attrition from treatment programs for abusive men J Fam Violence 2001 16 131 149 10.1023/A:1011106902465

[B31] Stalans LJ Seng M Identifying subgroups at high risk of dropping out of domestic batterer treatment Int J Offender Ther Comp Criminol 2007 51 151 169 10.1177/0306624X06290204 17412821

[B32] Magill M Ray LA Cognitive-behavioral treatment with adult alcohol and illicit drug users: a meta-analysis of randomized controlled trials J Stud Alcohol Drugs 2009 70 516 527 19515291 10.15288/jsad.2009.70.516 PMC2696292

[B33] Soyka M Schmidt P Outpatient alcoholism treatment – 24-month outcome and predictors of outcome Subs Abuse Treat Prev Policy 2009 4 15 serial online 10.1186/1747-597X-4-15 PMC2715386 19563659

[B34] Babcock JC Green CE Robie C Does batterers’ treatment work? A meta-analytic review of domestic violence treatment Clin Psychol Rev 2004 23 1023 1053 10.1016/j.cpr.2002.07.001 14729422

[B35] Coulter M VandeWeerd C Reducing domestic violence and other criminal recidivism: effectiveness of multilevel batterers intervention program Violence Vict 2009 24 139 152 10.1891/0886-6708.24.2.139 19459395

[B36] Dillman DA Smyth JD Christian LM Internet, mail, and mixed-mode surveys: the tailored design method 2009 Hoboken: John Wiley & Sons, Inc

[B37] Timko C Policies and services in residential substance abuse programs J Subst Abuse 1995 7 43 50 7655311 10.1016/0899-3289(95)90305-4

[B38] Timko C Physical characteristics of residential psychiatric programs Am J Community Psychol 1996 24 173 192 10.1007/BF02511886 8712185

[B39] Timko C Moos RH Determinants of the treatment climate in psychiatric and substance abuse programs J Nerv Ment Dis 1998 186 96 103 10.1097/00005053-199802000-00005 9484309

[B40] Timko C Moos RH Outcomes of the treatment climate in psychiatric and substance abuse programs J Clin Psychol 1998 54 1137 1150 10.1002/(SICI)1097-4679(199812)54:8<1137::AID-JCLP12>3.0.CO;2-0 9840784

[B41] Henderson CE Taxman FS Young DW A rasch model analysis of evidence-based treatment practices used in the criminal justice system Drug Alcohol Depend 2008 93 163 175 10.1016/j.drugalcdep.2007.09.010 18029116 PMC2293644

[B42] Herbeck DM Hser Y Teruya C Empirically supported substance abuse treatment approaches: a survey of treatment providers’ perspectives and practices Addict Behav 2008 33 669 712 10.1016/j.addbeh.2007.12.003 PMC2373985 18207334

[B43] McLellan AT Meyers K Contemporary addiction treatment Biol Psychol 2004 56 764 770 10.1016/j.biopsych.2004.06.018 15556121

[B44] Humphreys K McLellan AT A policy-oriented review of strategies for improving the outcomes of services for substance use disorder patients Addiction 2011 106 2058 2066 10.1111/j.1360-0443.2011.03464.x 21631620

[B45] Carise D Brooks A Alterman A McLellan AT Hoover V Forman R Implementing evidence-based practices in community treatment programs: initial feasibility of a counselor “toolkit Subst Abuse 2009 30 239 243 10.1080/08897070903041194 19591060

[B46] McLellan AT Chalk M Bartlett J Outcomes, performance, and quality: what’s the difference? J Subst Abuse Treat 2007 32 331 340 10.1016/j.jsat.2006.09.004 17481456

[B47] Daly JE Power TG Gondolf EW Predictors of batterer program attendance J Interpers Violence 2001 16 971 991 10.1177/088626001016010001

[B48] Mbilinyi L Walker D Neighbors C Roffman R Zegree J Edleson J Murphy CM, Maiuro RD Motivating substance-involved perpetrators of intimate partner violence to seek treatment: a focus on fathers Motivational interviewing and stages of change in intimate partner violence 2009 New York: Springer Publishing Co. 181 197

[B49] Stanton MW Rutherford MK Hospital nurse staffing and quality of care 2004 Rockville: Agency for Healthcare Research and Quality

[B50] Olmstead TA Abraham AJ Martino S Roman PM Counselor training in several evidence-based psychosocial addiction treatments in private US substance abuse treatment centers Drug Alcohol Depend 2012 120 149 154 10.1016/j.drugalcdep.2011.07.017 21831536 PMC3275814

[B51] Flynn PM Brown BS Co-occuring disorders in substance abuse treatment: issues and prospects J Subst Abuse Treat 2008 34 36 47 10.1016/j.jsat.2006.11.013 17574791 PMC2200799

[B52] Bennett LW Substance abuse by men in partner abuse intervention programs: current issues and promising trends Violence Vict 2008 23 236 248 10.1891/0886-6708.23.2.236 18624092

[B53] Taxman FS Henderson CE Belenko S Organization context, systems change, and adopting treatment delivery systems in the criminal justice system Drug Alcohol Depend 2009 103S S1 S6 19423241 10.1016/j.drugalcdep.2009.03.003

[B54] Lehman WEK Fletcher BW Wexler HK Melnick G Organizational factors and collaboration and integration activities in criminal justice and drug abuse treatment agencies Drug Alcohol Depend 2009 103S S65 S72 19307068 10.1016/j.drugalcdep.2009.01.004

[B55] McCarty D Chandler RK Understanding the importance of organizational and system variables on addiction treatment services within criminal justice settings Drug Alcohol Depend 2009 103S S91 S93 19356862 10.1016/j.drugalcdep.2009.03.001

[B56] Weisner C Mertens J Parthasarathy S Moore C Integrating primary medical care with addiction treatment: a randomized controlled trial JAMA 2001 286 1715 1723 10.1001/jama.286.14.1715 11594896 PMC3056510

[B57] Fox HC Alexrod P Paliwal P Sleeper J Sinha R Difficulties in emotional regulation and impulse control during cocaine abstinence Drug Alcohol Depend 2007 89 298 301 10.1016/j.drugalcdep.2006.12.026 17276626

[B58] Fox HC Hong KA Sinha R Difficulties in emotion regulation and impulse control in recently abstinent alcoholics compared with social drinkers Addict Behav 2008 33 388 394 10.1016/j.addbeh.2007.10.002 18023295

[B59] O’Farrell TJ Murphy CM Stephan SH Fal-Stewart W Murphy M Partner violence before and after couples-based alcoholism treatment for male alcoholic patients: the role of treatment involvement and abstinence J Cons Clin Psychol 2004 72 202 217 10.1037/0022-006X.72.2.202 15065955

[B60] O’Farrell TJ Van Hutton V Murphy CM Domestic violence before and after alcoholism treatment: a two-year longitudinal study J Stud Alcohol 1999 60 317 321 10371258 10.15288/jsa.1999.60.317

[B61] Brannen SJ Rubin A Comparing the effectiveness of gender-specific and couples groups in a court-mandated spouse abuse treatment program Res on Soc Work Practice 1996 6 405 424 10.1177/104973159600600401

[B62] McCollum EE Stith SM Couples treatment for interpersonal violence: a review of outcome research literature and current clinical practices Violence Vict 2008 23 187 201 10.1891/0886-6708.23.2.187 18624089

[B63] Johnson MP Leone JM The differential effects of intimate terrorism and situational couple violence J Fam Issues 2005 26 322 349 10.1177/0192513X04270345

[B64] Johnson MP Peters HE, Dush CMK Differentiating among types of domestic violence: Implications for healthy marriages Marriage and family: perspectives and complexitites 2009 New York: Columbia University Press 281 297

[B65] Dobkin P De Civita M Paraherakis A Gill K The role of social support in treatment retention and outcomes among outpatient adult substance abusers Addiction 2002 97 347 356 10.1046/j.1360-0443.2002.00083.x 11964111

[B66] Laudet AB Stanick V Sands B What could the program have done differently? A qualitative examination of reasons for leaving outpatient treatment J Subst Abuse Treat 2009 37 182 190 10.1016/j.jsat.2009.01.001 19339133 PMC2716417

[B67] McGovern MP Xie H Segal SR Siembab L Drake RE Addiction treatment services and co-occurring disorders: prevalence estimates, treatment practices, and barriers J Subst Abuse Treat 2006 31 267 275 10.1016/j.jsat.2006.05.003 16996389

[B68] Murphy C Maiuro R Motivational interviewing and stages of change in intimate partner violence 2009 New York: Springer

